# Why “covering all” in the DOTS program is not necessarily “all being covered” by the DOTS program

**DOI:** 10.4103/0970-2113.71975

**Published:** 2010

**Authors:** Lancelot Mark Pinto, Zarir Farokh Udwadia

**Affiliations:** *Department of Pulmonary Medicine, P.D. Hinduja National Hospital and Medical Research Centre, Mumbai - 400 016, India. E-mail: lance.pinto@gmail.com*

Sir,

We read with interest the Editorial by Paliwal R titled “Can DOTS improve treatment-seeking behavior of TB patients”.[1] The article raises several valid and relevant concerns, and we would like to share the results of a study we conducted at P. D. Hinduja National Hospital and Medical Research Centre that further echo these concerns and highlight the need for novel and innovative strategies to improve TB control in India.[2]

We administered a structured interview schedule to 200 consecutive adult TB patients with the aim of comprehending their awareness of the DOTS program and their preference for private healthcare. Only 30 of 200 patients (15%) were aware of the DOTS program. After being explained what directly observed therapy was, 136 patients (68%) found this form of treatment unacceptable. 183 patients (91.5%) preferred buying the drugs themselves rather than visiting a DOTS center. 90 patients (45%) were not prepared to be observed while swallowing their TB drugs, finding it an intrusion of privacy.

The success of the DOTS program in India is indubitable. However, this success and universal access is not commensurate with a universal preference for the program. A total of 50-70% of Indian TB patients continue to choose private healthcare,[3] and unless these patients are attracted to the program, they will remain there only.

Our study looked at potential means of attracting patients to the program based on the responses to our questionnaire. Awareness of the DOTS program as a viable and successful option for their treatment would be the first step, and as rightly pointed out by Paliwal R, mass media campaigns are the need of the hour to bring forth this awareness. The perceived image of government services was also poor, and this needs to be addressed to attract patients to the public sector. Public-private mixes (PPM’s) would be a step in the right direction, and successes of existing programs augur well for the model being used for successful TB control.[4] A factor that has been constantly stressed upon as being important to success, and yet often debated is directly observed treatment (DOT). Our patients found it cumbersome, and considered it as an intrusion of their privacy, and alternative options such as family member-observed DOT need to be explored.[5] It is the need of the hour to move from a didactic to a collaborative system of care to ensure compliance.

Despite achieving complete coverage of the population in 2006, tuberculosis continues to be a major challenge to the healthcare system in India, and unless we think of novel strategies to alter health-seeking behaviors and improve compliance while capitalizing on the early gains, the control of the disease might hit a roadblock, and the gains may plateau.
